# MULAN: multimodal protein language model for sequence and structure encoding

**DOI:** 10.1093/bioadv/vbaf117

**Published:** 2025-05-20

**Authors:** Daria Frolova, Marina Pak, Anna Litvin, Ilya Sharov, Dmitry Ivankov, Ivan Oseledets

**Affiliations:** Ligand Pro, Moscow 121205, Russia; Center for Artificial Intelligence Technology, Skolkovo Institute of Science and Technology, Moscow 121205, Russia; Ligand Pro, Moscow 121205, Russia; Center for Molecular and Cellular Biology, Skolkovo Institute of Science and Technology, Moscow 121205, Russia; Ligand Pro, Moscow 121205, Russia; Center for Molecular and Cellular Biology, Skolkovo Institute of Science and Technology, Moscow 121205, Russia; Belozersky Institute of Physico-Chemical Biology, Lomonosov Moscow State University, Moscow 119992, Russia; Ligand Pro, Moscow 121205, Russia; Center for Artificial Intelligence Technology, Skolkovo Institute of Science and Technology, Moscow 121205, Russia; Ligand Pro, Moscow 121205, Russia; Center for Molecular and Cellular Biology, Skolkovo Institute of Science and Technology, Moscow 121205, Russia; Ligand Pro, Moscow 121205, Russia; Center for Artificial Intelligence Technology, Skolkovo Institute of Science and Technology, Moscow 121205, Russia; AIRI, Moscow 105064, Russia

## Abstract

**Motivation:**

Most protein language models (PLMs) produce high-quality representations using only protein sequences. However, incorporating known protein structures is important for many prediction tasks, leading to increased interest in structure-aware PLMs. Currently, structure-aware PLMs are either trained from scratch or add significant parameter overhead for the structure encoder.

**Results:**

In this study, we propose MULAN, a MULtimodal PLM for both sequence and ANgle-based structure encoding. MULAN has a pre-trained sequence encoder and an introduced parameter-efficient Structure Adapter, which are then fused and trained together. Based on the evaluation of nine downstream tasks, MULAN models of various sizes show a quality improvement compared to both sequence-only ESM2 and structure-aware SaProt. The main improvements are shown for the protein–protein interaction prediction (up to 0.12 in AUROC). Importantly, unlike other models, MULAN offers a cheap increase in structural awareness of protein representations because of the finetuning of existing PLMs instead of training from scratch. We perform a detailed analysis of the proposed model and demonstrate its awareness of the protein structure.

**Availability and implementation:**

The implementation, training data, and model checkpoints are available at https://github.com/DFrolova/MULAN.

## 1 Introduction

Proteins play a pivotal role in nearly all biological functions (Finkelstein and Ptitsyn 2016). Comprising 20 distinct amino acids as unbranched heteropolymers, the specific sequence of these amino acids determines the complex three-dimensional (3D) structure of the protein (Anfinsen *et al.* 1961). Subsequently, this 3D configuration governs the protein’s function (Finkelstein and Ptitsyn 2016). Advances in genome sequencing have significantly increased the availability of protein data, providing a vast resource for understanding the molecular basis of life. Modern machine learning techniques can enhance our understanding of protein sequences, benefiting fields like drug discovery, protein design, and biotechnology.

The abundance of protein sequences and their text-like nature made it possible to apply natural language processing techniques to proteins. It is tempting to expect that protein sequence information alone would be sufficient for large protein language models (PLMs) to infer protein structure and function. Recently, large PLMs, such as ProtTrans (Elnaggar *et al.* 2021), ESM2 (Lin *et al.* 2022), and Ankh (Elnaggar *et al.* 2023), have made remarkable progress in protein representation learning, surpassing previous approaches across various downstream tasks. However, it appears that the representation abilities of sequence-only PLMs are limited and some kind of structural information should be encoded directly into the PLM. This limitation is represented by a significantly better performance (Lin *et al.* 2023) of structure-infused AlphaFold2 (Jumper *et al.* 2021) compared to sequence-only ESMFold (Lin *et al.* 2023).

Recent advancements like AlphaFold2 (Jumper *et al.* 2021) have made structural information about numerous proteins readily available. This has led to the development of structural protein language models (SPLMs) such as SaProt (Su *et al.* 2023) and ESM3 (Hayes *et al.* 2024), which incorporate structural knowledge and outperform sequence-only PLMs. However, these SPLMs require training from scratch and often involve large structure encoders, leading to increased parameter and computational overhead. This can limit their use in high-throughput applications like drug discovery and protein design.

**Figure 1.**
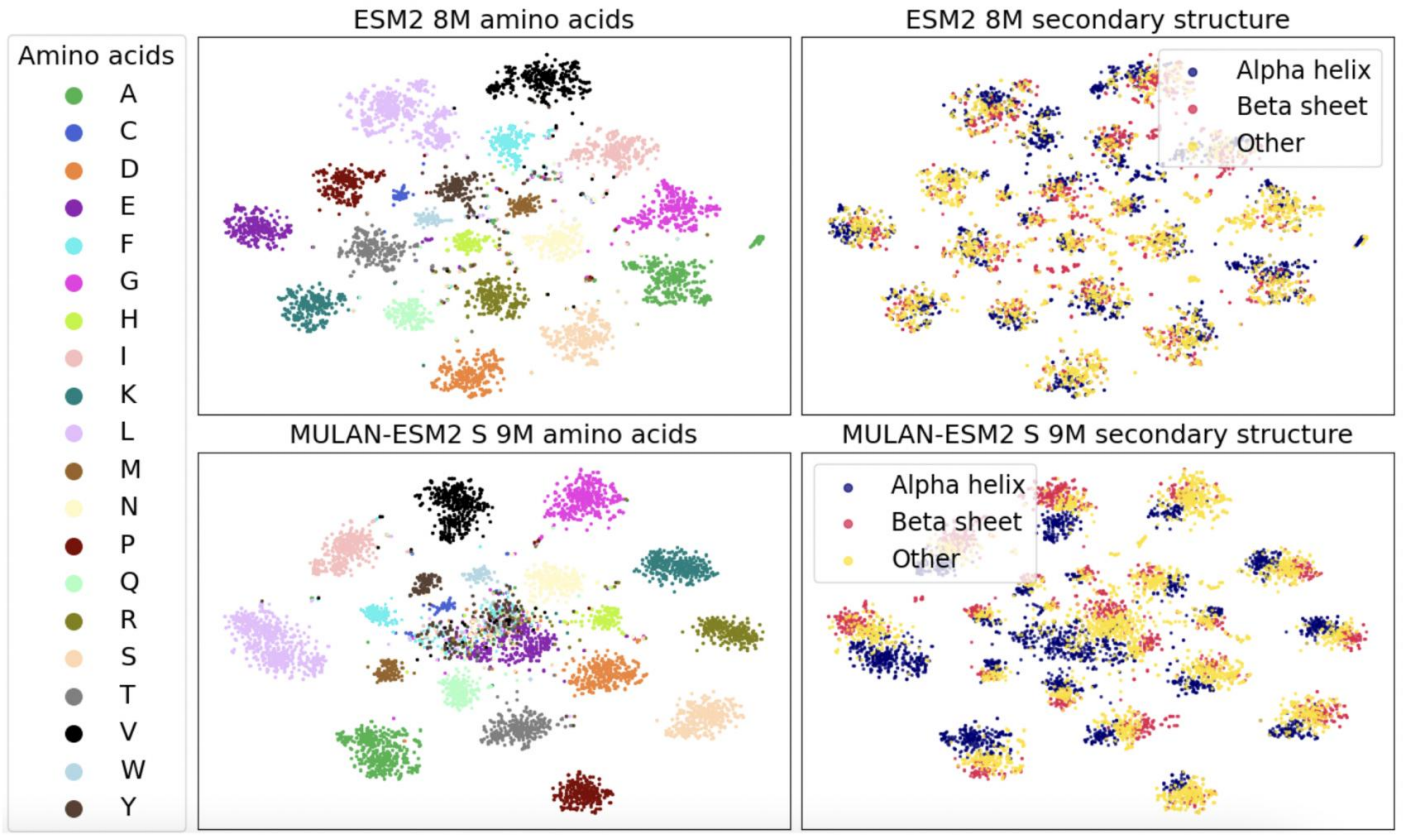
Visualization of residue embeddings of MULAN-ESM2 S 9M and ESM2 8M on CASP12 dataset. We use different colors for amino acid residue types (left) and for the three states of secondary structure (right). The details of the experiment are presented in Section 3.3.2 in Section 3.

In our study, we present a simple yet effective structure-aware PLM that is computationally and parameter-efficient and does not require training from scratch. Our main contributions are:

- We introduce MULAN, a MULtimodal PLM for both sequence and ANgle-based structure processing. We propose the Structure Adapter, a lightweight MULAN module that uses residue torsion angles to represent the protein structure. Our model can work on top of existing PLMs through PLM finetuning, so it offers a cheap increase of structural awareness due to avoiding training from scratch.
- We evaluate the obtained protein representations on a wide range of downstream tasks. We show that adding MULAN to ESM2 and SaProt of various sizes improves the downstream task performance. The main improvements are shown for the protein–protein interaction prediction (up to 0.12 in AUROC).
- Extensive ablation studies confirm MULAN’s effectiveness and structural awareness (see Section 3.2 and Fig. 1).

## 2 Methods

### 2.1 MULAN architecture

#### 2.1.1 Structural information

**Figure 2.**
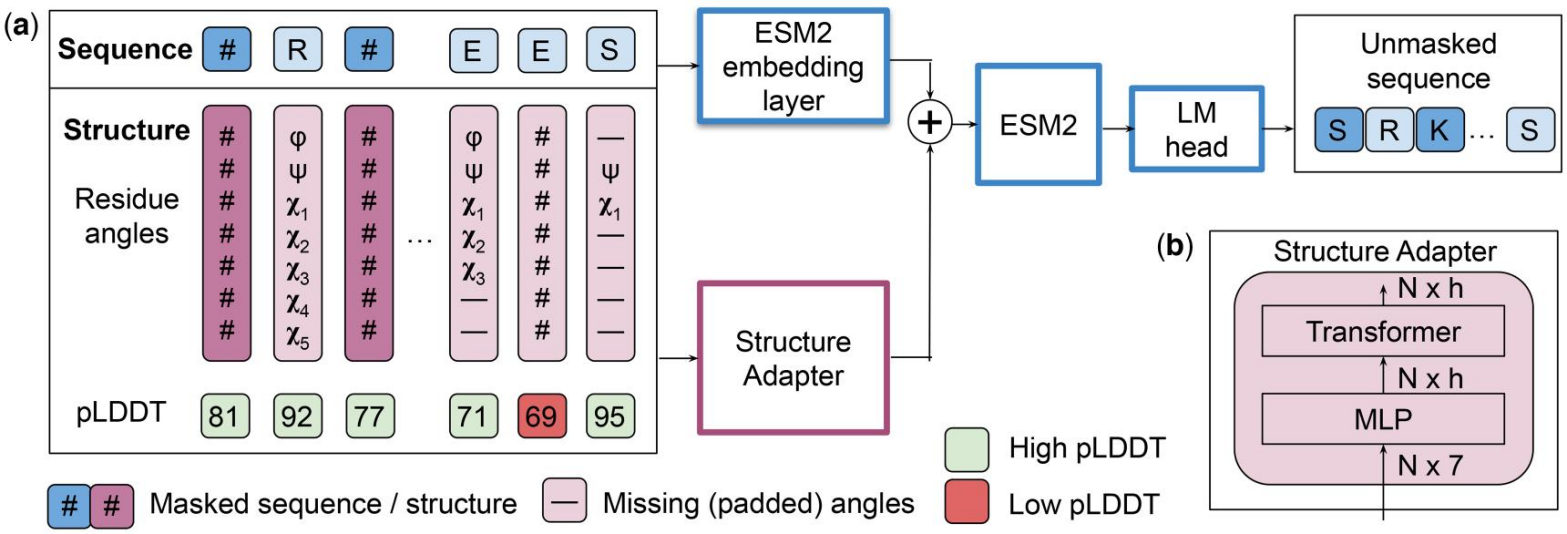
MULAN architecture. (a) MULAN processes sequence inputs with the ESM2 embeddings module, while structure inputs are passed to the Structure Adapter. Both sequence and structure embeddings are summed up and passed to the ESM2 model, which is then finetuned. Sequence-only ESM2 modules (blue) are initialized from the pre-trained ESM2 checkpoint. Structure processing modules are shown in pink. (b) The architecture of the Structure Adapter.

In this study, we propose MULAN, a MULtimodal encoder PLM for both sequence and ANgle-based structure processing. MULAN uses the pre-trained base PLM and has the Structure Adapter — a module we introduce to incorporate the knowledge about the protein structure (see Fig. 2a). In our experiments, we use ESM2 architecture, initializing the base PLM from ESM2 or SaProt models. However, MULAN can be based on other PLMs.

We use the information about the protein backbone torsion angles, which are conventionally called ϕ and ψ, to ensure the protein backbone flexibility. We also use all amino acid residue side chain torsion angles, which are conventionally called $\chi_{i}$ (up to five χ angles for amino acid arginine) and provide flexibility of the residues’ side chains. Missing χ angles together with undefined terminal ϕ and ψ angles are filled with the reserved padding value, which results in an angle vector $\left[\phi ,\psi ,\chi_{1},\chi_{2},\chi_{3},\chi_{4},\chi_{5}\right]$ for each residue. The residue torsion angles are rotation- and translation-invariant; thus, they are easy to use inside a transformer model.

#### 2.1.2 The Structure Adapter

The proposed Structure Adapter is used to support the multimodality of our model and to fuse structural information with the sequence-only PLM. It consists of an MLP that projects residue angles to embeddings with dimension *h*, followed by a Transformer layer with rotary positional embeddings (Su *et al.* 2024). For a protein of length *N*, the Structure Adapter returns an N×h angle embedding, or a structure bias. Finally, angle embeddings are added to initial ESM2’s residue embeddings. The resulting structure-aware residue embeddings are then passed through the ESM2 model. This lightweight design maintains efficiency while incorporating structural information.

### 2.2 Training procedure and structure masking

We initialize the base model with a pre-trained ESM2 checkpoint and then finetune it with additional structural information. We utilize the same masked language modeling objective (MLM) (Devlin *et al.* 2018) as ESM2 for sequences, randomly masking 15% of tokens in a batch. A similar masking strategy is applied to the Structure Adapter. Residue angles are masked or replaced jointly with the corresponding residue letters; 80% of the time, angles are masked with a pre-defined mask value, which are then passed to the Structure Adapter; 10% of the time, the angle vector is replaced by a random angle vector from the same protein, while in the rest of the cases, residue angles remain unchanged. Note that both reserved padding and masking angle values can have arbitrary values with absolute values higher than π to not mix with real angle values (we take 4 and −4).

For each residue, AlphaFold2 provides a confidence score with a [0−100] scale — predicted local distance difference test score (pLDDT) (Mariani *et al.* 2013). We observed that passing low-confidence predictions into the model worsens its performance (see Section 3.1). Therefore, residue angle vectors with pLDDT <70 are considered low confidence and are masked (see Fig. 2).

### 2.3 Training details

We use protein structures for Swiss-Prot proteins from AlphaFold2 Protein Structure Database (Varadi *et al.* 2022) for the pre-training stage (503,724 structures), excluding those shorter than 30 amino acids. The final set comprises 501,348 proteins. Following ESM (Rives *et al.* 2021), we randomly selected the validation set of 5000 proteins. MULAN models are initialized from corresponding pre-trained checkpoints: ESM2 8M for MULAN S (9M), ESM2/SaProt 35M for MULAN M (37M), and ESM2/SaProt 650M for MULAN L (652M). The extended training details and hyperparameters are detailed in Supplementary 1, available as supplementary data at *Bioinformatics Advances* online.

### 2.4 Downstream tasks

We evaluate MULAN on nine main downstream tasks and the secondary structure prediction task as a structural awareness check. Tasks include binary and 10-class Localization, Thermostability, Metal Ion Binding, Gene Ontology (GO), HumanPPI (from SaProt setup), Fluorescence (from Ankh setup), and Secondary structure [from TAPE benchmark (Rao *et al.* 2019)]. Details are in Supplementary 2.1 and Table 1.

**Table 1.** Performance gains induced by adding MULAN to ESM2 and SaProt models compared to the initial models used for MULAN initialization.^a^

Model name	Localization 10-cl./binary	Thermo-stability	Fluore scence	Metal Ion Binding	HumanPPI	GO CC/MF/BP
	acc ↑/AUC ↑	SCC ↑	SCC ↑	AUC ↑	AUC ↑	$F_{\text{max}}$ ↑
ESM2 8M	0.722 / **0.948**	0.657	0.585	0.739	0.657	**0.481** / 0.532 / 0.400
MULAN-ESM2 S	**0.732** / **0.948**	**0.674**	**0.596**	**0.814**	**0.717**	**0.481** / **0.583**/ **0.423**

ESM2 35M	0.760 / **0.963**	0.678	**0.592**	**0.783**	0.668	0.492 / 0.608 / 0.442
MULAN-ESM2 M	**0.764** / **0.963**	**0.682**	0.584	0.771	**0.787**	**0.504** / **0.628**/ **0.453**

SaProt AF 35M	0.767 / 0.960	**0.695**	0.634	0.784	0.703	**0.499** / **0.633** / 0.440
MULAN-SaProt M	**0.769** / **0.962**	**0.695**	**0.638**	**0.797**	**0.766**	0.496 / 0.632 / **0.444**

ESM2 650M	**0.818** / **0.968**	0.691	0.595	**0.786**	0.748	**0.516** / **0.676** / **0.479**
MULAN-ESM2 L	0.815 / **0.968**	**0.702**	**0.614**	0.766	**0.847**	0.515 / **0.676** / **0.479**

SaProt AF 650M	0.840 / **0.972**	**0.711**	**0.668**	0.779	0.712	**0.535** / **0.661** / **0.476**
MULAN-SaProt L	**0.841** / **0.972**	0.709	0.667	**0.798**	**0.766**	0.530 / 0.660 / 0.468

a The quality is measured on nine downstream tasks. We report average results across five random seeds used to initialize downstream models. We indicate the best results for each pair in bold. Statistically significant improvements with *P*-value <0.05 are underlined.

AlphaFold2 structures for proteins from the datasets were retrieved from AlphaFold2 DB by UniProt accession number if available. PDB IDs were mapped to UniProt accession numbers and retrieved from AlphaFold2 DB. If no protein identifier was available or there was no AlphaFold2 model for the UniProt accession number in the database, the protein was modeled by the standalone version of AlphaFold2. For all described datasets, we keep the original data splits. For the Secondary structure prediction, we use original experimental PDB structures for evaluation. The description of datasets and processing steps is detailed in Supplementary 2.1.

To evaluate binary classification tasks, we use the area under the ROC curve (AUC); for multiclass classification, we use accuracy; for multilabel GO annotation task, we follow (Gligorijević *et al.* 2021) and use $F_{\text{max}}$ score; and for the regression tasks, we measure Spearman’s correlation coefficient (SCC).

### 2.5 Downstream task evaluation

To evaluate the performance of SPLMs, we extract protein embeddings from the last layer of a model. For protein-level tasks, the average pooling of embeddings is done. For the downstream model, take the Light Attention architecture (Stärk *et al.* 2021), which was designed to work with protein embeddings and shows better results than an MLP. The downstream model architecture is detailed in Supplementary 2.2.

For each downstream task and PLM, we train the downstream model and optimize hyperparameters using validation metrics. We set a fixed grid of hyperparameters for all downstream tasks (see Supplementary 2.3). We use AdamW optimizer ($\beta_{1}=0.9$, $\beta_{2}=0.999$) with a fixed learning rate (in most setups it is $5\cdot 10^{-5}$) and batch size 8192. Models are trained for 200 epochs, selecting the best checkpoint based on validation performance.

## 3 Discussion

### 3.1 Results

As baseline models for comparison, we take sequence-only PLMs [ProteinBert (Brandes *et al.* 2022), ESM2 and Ankh]; structure-aware SPLMs [ProstT5 (Heinzinger *et al.* 2023), SaProt and ESM3]; hybrid sequence-structure models [S-PLM (Wang *et al.* 2023) and PST (Chen *et al.* 2024)], which are top-performing to the best of our knowledge. We do not compare to ESM-GearNet (Zhang *et al.* 2023), because SaProt has reported to be better (Su *et al.* 2023), while LM-GVP (Wang *et al.* 2022) and GearNet (Zhang *et al.* 2023) were surpassed by ESM-GearNet (Zhang *et al.* 2023). Another hybrid model DeProt (Li *et al.* 2024) does not have implementation available. We train MULAN on top of two model families: ESM2 and SaProt AF of various sizes. The quality of the protein representations on the considered downstream tasks is reported in Table 2. Note that we report results for models of different sizes in separate parts of the table and highlight the best results in bold also separately according to the model size. Our main findings are discussed below.

**Table 2.** Comparison of the performance of various PLMs and SPLMs on nine main downstream tasks.^a^

Model name	Localization	Thermo-	Fluore	Metal Ion	HumanPPI	GO
	10-cl./binary	stability	scence	Binding		CC/MF/BP
	acc ↑/AUC ↑	SCC ↑	SCC ↑	AUC ↑	AUC ↑	$F_{\text{max}}$ ↑
**Small models**						
ProteinBert 16M	0.692 / 0.952	0.636	**0.610**	0.754	0.687	0.447 / 0.542 / 0.415
ESM2 8M	0.722 / **0.948**	0.657	0.585	0.739	0.657	0.418 / 0.532 / 0.400
MULAN-ESM2 S	**0.732** / **0.948**	**0.674**	0.596	**0.814**	**0.717**	**0.481** / **0.583** / **0.423**

**Medium models**						
ESM2 35M	0.760 / **0.963**	0.678	0.592	0.783	0.668	0.492 / 0.608 / 0.442
MULAN-ESM2 M	0.764 / **0.963**	0.682	0.584	0.771	**0.787**	**0.504** / 0.628 / **0.453**
SaProt AF 35M	0.767 / 0.960	**0.695**	0.634	0.784	0.703	0.499 / **0.633** / 0.440
MULAN-SaProt M	**0.769** / 0.962	**0.695**	**0.638**	**0.797**	0.766	0.496 / 0.632 / 0.444

**Large models**						
Ankh base 450M	0.804 / 0.966	0.703	0.630	0.837	0.758	0.510 / 0.686 / 0.495
Ankh large 1.2B	0.806 / 0.954	0.668	0.638	0.787	0.738	0.517 / **0.692** / **0.501**
ProstT5 1.2B	0.773 / 0.954	0.693	0.633	0.805	0.663	0.518 / 0.687 / 0.484
PST 1.1B	0.820 / 0.965	0.689	0.618	0.831	0.809	0.526 / 0.676 / 0.475
S-PLM 704M	0.796 / 0.950	0.678	0.584	0.767	0.731	0.486 / 0.671 / 0.466
ESM3 1.4B	0.751 / 0.951	0.695	0.663	**0.846**	0.704	0.512 / 0.673 / 0.471

ESM2 650M	0.818 / 0.968	0.691	0.595	0.786	0.748	0.516 / 0.676 / 0.479
MULAN-ESM2 L	0.815 / 0.968	0.702	0.614	0.766	**0.847**	0.515 / 0.676 / 0.479
SaProt AF 650M	0.840 / **0.972**	**0.711**	**0.668**	0.779	0.712	**0.535** / 0.661 / 0.467
MULAN-SaProt L	**0.841** / **0.972**	0.709	0.667	0.798	0.766	0.530 / 0.660 / 0.468

a The table is split into sections according to model sizes. We indicate the best results in bold for each section separately. For large models second best results are underlined.

#### 3.1.1 Adding MULAN is beneficial for different PLMs

First of all, we aim to highlight the downstream performance gains introduced by MULAN when applied to both ESM2 and SaProt models. These improvements are shown in Table 1: we report pairs of MULAN models compared to the base PLMs used for MULAN initialization. We report average results across 5 random seeds used to initialize downstream models. The best results for each pair are indicated in bold. Statistically significant improvements with the *p*-value <0.05 are underlined. We also report the standard deviations for each experiment in Supplementary 6. The results demonstrate that MULAN is effective for both small, medium, and large models. We show that the performance of both considered PLMs was mostly improved by adding the proposed Structure Adapter.

MULAN shows strongest improvements when applied to the small ESM2 model, significantly increasing the quality of protein function prediction (GO, MF, and BP) as well as protein interaction prediction (Metal Ion Binding and HumanPPI). This fact indicates that structure-related downstream tasks benefit from the structural input. For medium and large models, adding MULAN is mostly showing better results compared to the initial models. Especially high-performance gains, which are statistically significant, are observed for the HumanPPI task, further highlighting the dependence of protein–protein binding patterns on their structure.

Furthermore, we show that MULAN-ESM generally offers higher performance gains compared to MULAN-SaProt. We suppose that it is because SaProt is already a structure-aware model, while ESM2 is sequence-only. We show that this simple strategy can enhance PLM performance for all considered models. We expect that MULAN can be applied to any large PLM (for example, Ankh), further improving their performance by a computationally-efficient fine-tuning.

#### 3.1.2 MULAN further boosts structure-aware SaProt

Even though SaProt already uses protein structure information, MULAN-SaProt enhances SaProt’s performance, especially in protein interaction tasks, while maintaining baseline performance elsewhere. This suggests that Foldseek’s structural encoding can be complemented by MULAN’s Structure Adapter for better protein representation. The Structure Adapter’s success suggests potential for further improvements in leveraging 3D structural information for protein representation.

#### 3.1.3 MULAN works on par with other SPLMs

While MULAN’s focus is on efficient enhancement of existing PLMs rather than state-of-the-art performance, we compare it with relevant baselines in Table 2. MULAN-ESM2 S shows the best results compared to small ESM2 8M and even twice larger ProteinBert 16M model. To the best of our knowledge, in the medium-sized models, there are only already considered ESM2 and SaProt, so we have discussed them earlier. As for large models, there is no clear superiority of one model over others among baselines. There are downstream tasks where each model can show good results. However, MULAN performs strictly better than S-PLM and is comparable to ProstT5, ESM3, and PST (there are approximately half of the tasks better in MULAN L).

#### 3.1.4 MULAN offers cheap structural awareness

Remarkably, MULAN achieves its improvements efficiently: it requires only brief finetuning (up to 3 days on one GPU) instead of months of training for models like ProstT5, ESM3, and SaProt. Thus, MULAN is very computationally friendly in terms of training time and applying it to new models. Secondly, MULAN adds the minimal parameter overhead. It adds just 0.3% parameter overhead to ESM2 L (652M parameters), compared to PST’s 69% and S-PLM’s 13.8%. These additional large structure encoders significantly decrease the inference speed and require more powerful GPUs, which is undesirable in practical scenarios (see Supplementary 5). Despite its lightweight design, MULAN outperforms S-PLM and matches PST’s performance, showing that a minimal approach can work comparably to larger models.

### 3.2 Ablation study

Below, we present the main ablation results, while detailed computational efficiency analysis, ablation studies and the analysis of hyperparameter are available in Supplementary 3.

#### 3.2.1 The importance of the Structure Adapter

To isolate the Structure Adapter’s impact, we finetune the pure ESM2 8M on our training dataset (ESM2 + finetune experiment) to show that our training dataset and the finetuning procedure itself do not lead to a significant performance boost on downstream tasks. The results demonstrate that finetuning of ESM2 on our data is not enough, and the main contribution to the performance improvements is done by the Structure Adapter (see ESM2 vs ESM2 + finetune vs MULAN-ESM2 experiments in Table 3).

**Table 3.** Ablation study of the training pipeline for ESM2 8M and MULAN-ESM2 S.^a^

Model name	Localization	Thermo-	Fluore	Metal Ion	HumanPPI	GO
	10-cl./binary	stability	scence	Binding		CC/MF/BP
	acc ↑/AUC ↑	SCC ↑	SCC ↑	AUC ↑	AUC ↑	$F_{\text{max}}$ ↑
**ESM2 8M**	0.722 / **0.948**	0.657	0.585	0.739	0.657	0.481 / 0.532 / 0.400
ESM2 + finetune	0.715 / 0.946	0.679	0.576	0.743	0.728	0.476 / 0.545 / 0.410

**MULAN-ESM2 S**	**0.732** / **0.948**	0.674	**0.596**	**0.814**	0.717	0.481 / **0.583** / **0.423**
without pLDDT	0.712 / 0.937	**0.684**	0.582	0.760	0.738	**0.496** / 0.579 / 0.420
from scratch	0.668 / 0.922	0.663	0.550	0.709	**0.765**	0.461 / 0.514 / 0.373

a The first section corresponds to ESM2 8M results without the Structure Adapter, while the second section to MULAN-ESM2 S. The best results are shown in bold and second best are underlined.

#### 3.2.2 Masking structural inputs with pLDDT

We show that it is useful to mask uncertain residues in AlphaFold2 structure models (with pLDDT >70) before passing them to the Structure Adapter: see MULAN vs MULAN without pLDDT masking experiments in Table 3. This trick is beneficial for most downstream tasks, likely due to the noise reduction in input angles.

#### 3.2.3 Starting from the pre-trained model is necessary

Finetuning pre-trained ESM2 is more effective than training MULAN from scratch. Adding structural information while finetuning preserves ESM2’s pre-trained knowledge while adapting the model to handle structural inputs. We applied the same training procedure to randomly initialized MULAN (see Table 3: MULAN vs MULAN from scratch), and the obtained results are much worse compared even to ESM2 8M. This fact indicates the need for much more time for training MULAN from scratch.

#### 3.2.4 Architecture ablation

Following SaProt and ProstT5 (Su *et al.* 2023, Heinzinger *et al.* 2023), we try to use Foldseek sequences to represent the structure in the same manner as the Structure Adapter does. Also, we experimented with structure features prediction heads and additional structure-related loss functions. As a result, we tried to add an additional Foldseek embedding layer or Contact and Angle prediction heads. According to our experiments, we did not notice a significant quality improvement compared to the base MULAN induced by these modifications (see Table 3). We explain these architectural modifications in detail and discuss the obtained results in Supplementary 3.1.

### 3.3 Secondary structure prediction

We aim to show the awareness and proper use of 3D structure by MULAN. For this purpose, we evaluate our model on the secondary structure prediction downstream task (see Table 4). We report results on CASP12 (Moult *et al.* 2018), TS115 (Yang *et al.* 2018), and CB513 (Cuff and Barton 1999) datasets. Initially, MULAN was trained using AlphaFold2 structures. It masks uncertain residue predictions based on the AlphaFold2 pLDDT score. We evaluate the quality of secondary structure prediction using the initial datasets with experimental structures. For such type of structures, pLDDT is not applicable, so we pass angle information for all residues into MULAN without masking.

**Table 4.** Comparison of the performance of PLMs on the secondary structure prediction task.^a^^a^

Model name	3-state, accuracy ↑			8-state, accuracy ↑		
	CASP12	TS115	CB513	CASP12	TS115	CB513
**Small models**						
ESM2 8M	0.732	0.798	0.765	0.602	0.677	0.623
MULAN-ESM2 S	**0.894**	**0.918**	**0.895**	**0.815**	**0.854**	**0.806**

**Medium models**						
ESM2 35M	0.752	0.828	0.809	0.619	0.707	0.669
MULAN-ESM2 M	0.886	0.901	0.877	0.789	0.814	0.769
SaProt AF 35M	0.900	0.924	0.910	0.805	0.848	0.817
MULAN-SaProt M	**0.905**	**0.927**	**0.912**	**0.807**	**0.852**	**0.818**

a The table is split into sections based on the model size. The best results for each section are in bold.

#### 3.3.1 Structural awareness of MULAN

According to the results from Table 4, MULAN models demonstrate the awareness of the protein secondary structure. They surpass similar-sized ESM2 models by a large margin. The same holds for large models, whose results are shown in Table 6 in Supplementary 4. We do understand that the correct information about the secondary structure can be derived from angle inputs as well as from the Foldseek tokens used by SaProt. Hence, this experiment is done to demonstrate that MULAN actively uses the 3D structure.

#### 3.3.2 Visualization

The quality of the separation of the PLM representations according to some physical or structural property can serve as evidence of the model’s physical and structural awareness. We perform a comparison of MULAN-ESM2 S and ESM2 8M representations according to the visual quality of the t-SNE (Van der Maaten and Hinton 2008) visualization. We plot residue-level embeddings from the last layer of both models on the CASP12 dataset (Moult *et al.* 2018). We highlight in color different amino acid residue types on the left and secondary structure types (3 states) on the right (see Fig. 1). On the one hand, MULAN shows better secondary structure awareness than ESM2: for most amino acid clusters three secondary structure types are separated, while for ESM2 they are mostly mixed. On the other hand, MULAN does not lose the initial knowledge about the amino acid properties gained from the ESM2 model. All these findings are in line with the pre-training strategy. ESM2 has sequences as inputs, so it aligns amino acid representations in separate clusters. MULAN has both sequences and structure inputs; therefore, its representations are aligned in both domains.

### 3.4 Related work

#### 3.4.1 Sequence-based models

Protein sequences are similar to human language: like letters are assembled into words that, in turn, form sentences, amino acids are chained into protein sequences that encode protein 3D structure which determines function. This resemblance makes it promising to apply best practices from the natural language processing field for solving protein-related tasks. Most PLMs are pre-trained with a masked language modeling (MLM) objective (Devlin *et al.* 2018): a part of the input sequence’s residues is randomly masked or replaced with other residues, and then the model aims to predict these corrupted tokens using the remaining sequence context. Models from the Transformer family (Heinzinger *et al.* 2019, Xiao *et al.* 2021, Rives *et al.* 2021, Brandes *et al.* 2022, Lin *et al.* 2023, Elnaggar *et al.* 2021, 2023) have made huge progress in the protein representation learning, among which ESM2 (Lin *et al.* 2023) and Ankh (Elnaggar *et al.* 2023) are currently showing the best results. They show high performance in various downstream tasks, for example, the prediction of protein secondary structure, residue contacts, sub-cellular localization, and the effect of mutation.

#### 3.4.2 Structure-informed models

The amino acid sequence solely defines the protein structure (Anfinsen *et al.* 1961), which, in turn, defines all protein properties, including its function. However, the sequence alone is not sufficient enough for PLMs to infer all information about the protein (Lin *et al.* 2023). Thus, attempts to infuse PLMs with structural context were made. To improve PLM’s capabilities, it was proposed to finetune ESM2 on the remote homology detection task (Zhang *et al.* 2024), which seems to implicitly incorporate protein structure-based features into the model, since the protein structure is more conserved than the protein sequence (Chothia and Lesk 1986).

Recently, the idea of using protein 3D structure directly during the model pre-training has been given a lot of attention from the research community. ProstT5 (Heinzinger *et al.* 2023), the first structure-augmented PLM, uses Foldseek, a special structural alphabet (van Kempen *et al.* 2024) (3Di) that describes the tertiary structure. As a result, each protein can be represented with either an amino acid sequence or a string of Foldseek letters of the same length that carries information about tertiary interactions. Incorporating that information into PLM was done by finetuning a sequence-only ProtT5 model to translate between the amino acid sequence and 3Di sequence to obtain structure-aware protein representations. Furthermore, even adding Foldseek encoding directly to the trained PLM embeddings without finetuning improves the protein-protein interaction prediction quality (Sledzieski *et al.* 2023). Another structure-informed PLM, SaProt (Su *et al.* 2023), uses the 3Di alphabet to encode the structure similarly to ProstT5. It represents each residue as a combination of amino acid and 3Di letters and is trained with MLM from scratch. This approach gives SaProt an improvement over ESM2 on various downstream tasks. Tan *et al.* (2024) suggest an adapter-based approach (SES-Adapter) that works with Foldseek sequences and residue secondary structure annotation. However, it is an approach for structure-aware downstream task tuning rather than a general-purpose PLM. It needs to be applied and trained for each downstream task separately and does not provide protein structure-aware embeddings for general use. As a result, SES-Adapter cannot be compared to MULAN. Recently, ESM3 (Hayes *et al.* 2024) has incorporated sequence, structure, and function modalities for the protein representation learning task as well as protein generation. Similarly to MULAN, they embed and fuse different modalities of the protein, but train a large PLM from scratch.

#### 3.4.3 Hybrid models

The graph-like tertiary structure of proteins gives ideas of infusing PLMs with structure information via Graph Neural Networks. In this setup, pre-training is left as MLM only (Mansoor *et al.* 2021, Zheng *et al.* 2023) or is augmented by Masked Structure Modeling task where not only parts of the sequence are masked but parts of the structure too (LM-GVP (Wang *et al.* 2022), GearNet (Zhang *et al.* 2023), and MIF (Yang *et al.* 2023)). In ESM-GearNet (Zhang *et al.* 2023), it was proposed to fuse the protein sequence and structure information from state-of-the-art PLMs with graph structure encoders (GearNet). The authors used various pre-training strategies including diffusion-based and reported a performance boost compared to ESM2 and GearNet on several downstream tasks. DeProt (Li *et al.* 2024) works similarly to ESM-GearNet. DeProt uses local protein structure around the residue to get the residue-level structure encoding. Chen *et al.* (2024) presented PST, an approach to combine a graph encoder and a PLM and to jointly train them to obtain structure-aware protein representations. PST modifies the self-attention mechanism of the underlying PLM and trains the whole model jointly. The graph encoder has as many parameters as the base PLM used. PST shows better results compared to relevant baselines, e.g. ESM2 and ESM-GearNet. Wang *et al.* (2023) presents S-PLM, a contrastive learning approach to jointly train protein sequence and structure encoders. A structure encoder is a Swin-Transformer that works on residue-residue contact matrices and is trained from scratch. S-PLM does not take protein structure explicitly during inference, relying only on the learned structure-aware representations.

## 4 Conclusion

In this paper, we propose MULAN, a novel multimodal 3D structure-aware protein language model for both sequence and structure processing. MULAN works on top of a pre-trained PLM and has the introduced lightweight Structure Adapter that processes residue dihedral angles. Our model finetunes the pre-trained PLM model offering a cheap incorporation of the knowledge about the protein structure into the model. Also, unlike other hybrid structure-aware models, the Structure Adapter of MULAN adds minor parameter overhead to the base PLM: 0.3% for large MULAN. We train MULAN models of various sizes and evaluate their protein representations on nine downstream tasks. For most of the downstream tasks, our model demonstrates an increase in performance compared to ESM2 and SaProt models, which were used for MULAN initialization. The most impressive results, which are statistically significant, were shown for the protein-protein interaction task. Additionally, MULAN demonstrates comparable performance to ESM3, ProstT5, and other PLMs considered in the study, while having a faster training or inference pipeline. Finally, we experimentally demonstrate MULAN’s structural awareness.

## Supplementary Material

vbaf117_Supplementary_Data
