# Supplementary material for: MULAN: multimodal protein language model for sequence and structure encoding
Source: Bioinform Adv. 2025 May 20;5(1):vbaf117. doi: 10.1093/bioadv/vbaf117 (PMC12452268; doi:10.1093/bioadv/vbaf117)
Supplement: vbaf117_Supplementary_Data [file vbaf117_supplementary_data.pdf]

MULAN: Multimodal Protein Language Model for Sequence and Structure Encoding

Supplementary information

Daria Frolova, Marina Pak, Anna Litvin, Ilya Sharov, Dmitry Ivankov, Ivan Oseledets

1. Training details

We used AdamW optimizer ($\beta_1 = 0.9$, $\beta_2 = 0.999$) [1] for all MULAN models. MULAN-ESM2 S was trained with the learning rate 10^{-4} during 20 epochs and with 12000 tokens per batch. The training process is run on 1 Tesla V100 GPU and lasts approximately 18 hours (375k steps).

Both medium and large models were trained using 1 Tesla H100 GPU for approximately 360k steps. For medium models, we used 32000 tokens per batch and the learning rate 10^{-5}, and it resulted in 65 epochs and 1.5 days. For large we took 10000 tokens per batch and the learning rate $5 \cdot 10^{-6}$, and it resulted in 20 epochs and 3 days.

During the pre-training stage, we randomly crop proteins that are longer than 1022 residues to the length of 1022. We follow ESM [2] and use dynamic batch size without the concatenation of proteins along the sequence dimension during training. Also, to fully utilize GPU and minimize the amount of padding, we use sorted batching with dynamic batch size as in [3]: we keep a fixed number of tokens in the batch and form the batch from proteins with similar lengths.

2. Downstream tasks

2.1. Downstream datasets

We follow SaProt and use their setup for protein Localization, Thermostability, Metal Ion Binding, GO, and HumanPPI. Localization prediction from DeepLoc dataset [4] has two tasks: classification of proteins into 2 and 10 categories, which both reported. For Thermostability prediction, the "Human-cell" split from FLIP benchmark [5] is used. It relies on human data from Meltom atlas [6]. Also, one of the considered downstream tasks is Metal Ion Binding: we predict whether there are metal ion-binding sites in the protein [7]. The prediction of protein-protein interaction for human proteins (HumanPPI) [8] is taken from PEER benchmark [9]. We predict GO terms [10] and use all three branches independently: Molecular Function (MF), Biological Process (BP), and Cellular Component (CC). GO annotation is a multilabel prediction task. For all listed downstream tasks we use data provided by [11], so all used AlphaFold protein structures are available in the AlphaFold database.

Fluorescence prediction is done based on the data of the fluorescence intensity of green fluorescent protein (GFP) mutants [12]. We follow the setup of Ankh evaluation and use the split from TAPE [13] benchmark. We built an AlphaFold structure of the wild-type GFP protein and used Rosetta relaxation protocol [14] for the generation of mutant 3D structures. GFP_wt sequence was taken from the original dataset [12]. The reference GFP structure was provided to Rosetta to build mutant structures. Since these are single mutants, their structures should not differ a lot from the structure of wild-type GFP, and simple relaxation is enough. Then, we used pLDDT scores from the initial GFP structure for training on all mutant proteins.

Moreover, we evaluate our model on the secondary structure prediction task which is taken from TAPE benchmark [13]. We report results on three test datasets: CASP12 [15], TS115 [16] and CB513 [17], both for 3-state and 8-state setups. For this task, only experimental structures are available, so we use them as an input to MULAN. For all experimental structures, we pass all residue angles without masking into the MULAN. This is done because of the absence and inapplicability of pLDDT to the experimental structures.

We summarize the information about all downstream tasks in Table 1.

2.2. Downstream model architecture

Downstream task prediction is done using the model with the Light Attention architecture [18], which was designed to work with protein embeddings and shows better results than an MLP. The only difference is that we extend it by adding two extra intermediate layers L_1 and L_2: $L_i = \text{Dropout}(\text{ReLU}(\text{BatchNorm}(\text{Linear}))) : \mathbb{R}^{h_{i-1}} \to \mathbb{R}^{h_i}$, where h_1 and h_2 are the model hyperparameters, and h_0 is the initial embedding dimension. They are added before the output Linear layer, which projects embeddings of size h_2 into the downstream task target dimension.

2.3. Downstream task hyperparameters

Here we present the grid used to select optimal hyperparameters for all downstream tasks (see 2). For the GO task, we have selected and fixed hyperparameters that perform well for all PLMs. We do not perform grid search because of the long time required for a single evaluation. Moreover, for GO it was optimal to increase the learning rate because of the much bigger output dimension in this task: up to 1943 classes for GO BP. For the HumanPPI task, the optimal learning rate differs from the base one due to the different nature of the task: we need to input two concatenated protein embeddings instead of one to the downstream model. Also, we reduced the grid for HumanPPI (take 0.2 dropout rate) to decrease the number of required computations. The batch size is equal to 8192 for all experiments, and the training time is 200 epochs, but we select the intermediate checkpoint with the best validation metric. Since we use the secondary structure prediction task only to show the structural awareness of the model, we fix hyperparameters for the downstream task evaluation for a faster model evaluation.

3. Ablation studies

In this section, we present all ablation experiments that were performed during the selection of the architecture and the

Table 1. Downstream tasks summary

Task name	Prediction level	Task type	Evaluation metric	Data split sizes train / valid / test
Localization (10-class)	protein	multiclass classification	accuracy	8,743 / 2,190 / 2,745
Localization (binary)	protein	binary classification	AUROC	5,477 / 1,336 / 1,731
Thermostability	protein	regression	SCC	5,056 / 639 / 1,336
Fluorescence	protein	regression	SCC	21,446 / 5,362 / 27,217
Metal Ion Binding	protein	binary classification	AUC	5,066 / 662 / 665
HumanPPI	protein pair	binary classification	AUC	26,317 / 234 / 180
GO CC	protein	multilabel classification	F_{max}	26,225 / 2,904 / 3,350
GO MF	protein	multilabel classification	F_{max}	26,225 / 2,904 / 3,350
GO BP	protein	multilabel classification	F_{max}	26,225 / 2,904 / 3,350
Secondary structure	residue	multiclass classification	accuracy	8,678 / 2,170 / 21; 115; 434

Table 2. Downstream task hyperparameters: learning rate (lr), dropout rate (dropout), intermediate representation sizes h_1 and h_2

Task name	lr	dropout	h_1	h_2
Localization (10-class)	$5 \cdot 10^{-5}$	$\{0.1, 0.2\}$	$\{1536, 1024, 512\}$	$\{512, 256\}$
Localization (binary)	$5 \cdot 10^{-5}$	$\{0.1, 0.2\}$	$\{1536, 1024, 512\}$	$\{512, 256\}$
Metal Ion Binding	$5 \cdot 10^{-5}$	$\{0.1, 0.2\}$	$\{1536, 1024, 512\}$	$\{512, 256\}$
HumanPPI	$5 \cdot 10^{-6}$	0.2	$\{1536, 1024, 512\}$	$\{512, 256\}$
GO CC / MF / BP	$5 \cdot 10^{-4}$	0.1	1536	768
Thermostability	$5 \cdot 10^{-5}$	$\{0.1, 0.2\}$	$\{1536, 1024, 512\}$	$\{512, 256\}$
Fluorescence	$5 \cdot 10^{-5}$	$\{0.1, 0.2\}$	$\{1536, 1024, 512\}$	$\{512, 256\}$
Secondary structure	$1 \cdot 10^{-4}$	0.1	1024	512

training procedure of MULAN as well as the hyperparameter tuning.

3.1. Alternative ways of presenting structural information to MULAN do not result in significant gains

There are many ways of representing the protein structure and infusing the structural information into the PLM. We have chosen to use the residue torsion angles as a model input. However, we have tried different approaches discussed below.

3.1.1. Foldseek as another structure input

Following SaProt and ProstT5, we try to use Foldseek sequences to represent the structure. We try to do it in the same manner as the Structure Adapter does. We add the Foldseek embedding layer, which is then finetuned together with ESM2. Also, we tried to combine both the Structure Adapter and the Foldseek embedding layer. The extended architecture of MULAN is shown in Figure 1.

3.1.2. Coordinates as another structure input

Additionally, we try to use xyz coordinates of C_α atoms to represent the structure. We treat them with another Structure Adapter that projects a 3-dimensional coordinate vector into an embedding of size h. Then, the resulting coordinate embedding is summed up with ESM embeddings and angle embeddings.

3.1.3. The objective function

MULAN is trained in the same way as ESM2 and most other PLMs: it uses the masked language modeling objective (MLM). One can use the knowledge about the protein structure not only as model input but also in the additional loss functions. We have experimented with two extra heads for the prediction of protein structure features, which were added to MULAN to increase its structural awareness. Firstly, we tried to restore masked angle inputs similarly to the original sequence MLM objective. This is done using the Angle Prediction Head, which has the same architecture as the ESM2 language modeling (LM) head except for the output dimension, which is 7, the number of residue angles. We use mean squared error (MSE) as a loss function.

Moreover, we use binarized pairwise distances between all residues in the form of a distance matrix as another source of structural data. For this purpose, we introduce a Contact Prediction Head. We binarize distances into 5 bins separated by the following distances: {5Å, 8Å, 16Å, 32Å}. Further, we predict $N \times N$ contact matrix based on the $N_{\text{heads}} * N_{\text{layers}} \times N \times N$ model attention weight tensor from all N_{layers} layers with N_{heads} attention heads. We use cross entropy (CE) loss and compute it only for residues with confident AlphaFold predictions to avoid training on noisy data. To sum up, we use the following objective function $\mathcal{L}$ during training:

$$\mathcal{L} = \mathcal{L}_{\text{CE}}(\text{sequence}) + \alpha\mathcal{L}_{\text{MSE}}(\text{angles}) + \beta\mathcal{L}_{\text{CE}}(\text{contacts}), \quad (1)$$

where α and β are floating point training hyperparameters. However, in the experiments that use structure features prediction heads, we utilize $\alpha = 5$ and $\beta = 0.5$ as they have shown the best performance on downstream tasks.

Similarly to the structural inputs, we compute both contact and angle prediction losses only on residues with the reliable AlphaFold structure with pLDDT not less than 70.

3.1.4. Architecture ablation study results

We have conducted an ablation study to identify the most important modules among those we have discussed earlier. We consider the Structure Adapter and Foldseek embedding layer as input modules that encode structure for MULAN. Also, we experiment with the Angle and Contact prediction

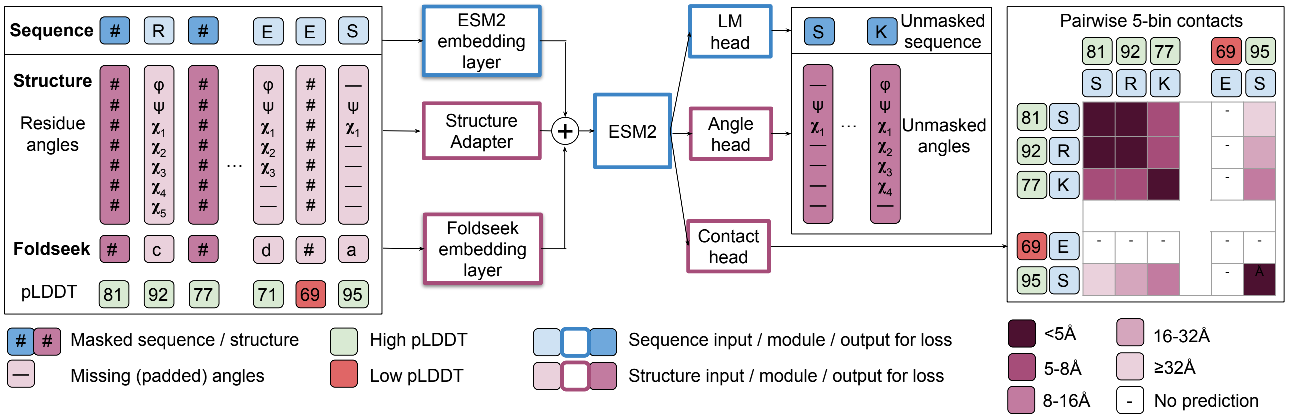

Fig. 1. The extended MULAN architecture. There is an extra Foldseek embedding layer and two output structure features prediction heads: Contact and Angle heads. Both sequence, structure, and Foldseek embeddings are summed up and passed to the ESM2 model, which is then finetuned. Sequence-only ESM2 modules are initialized from the pre-trained ESM2 checkpoint and are shown in blue. Structure processing modules are shown in pink.

Table 3. Ablation study of the training pipeline for ESM2 8M and MULAN-ESM2 S. The first section corresponds to ESM2 8M results without the Structure Adapter, while the second section – for MULAN-ESM2 S. The best results for each section are shown in bold, the second best are underlined

Model name	Localization 10-cl. / binary	Thermo-stability	Fluore scence	Metal Ion Binding	HumanPPI	GO CC / MF / BP
	acc ↑ / AUC ↑	SCC ↑	SCC ↑	AUC ↑	AUC ↑	F_{max} ↑
**ESM2 8M**	.722/**.948**	.657	.585	.739	.657	.481/.532/.400
ESM2 + Angle	.709/.942	.665	.562	.735	.740	.481/.540/.410
ESM2 + Contact	.706/.942	.675	.553	.703	.692	.477/.533/.406
**MULAN-ESM2 S**	.732/**.948**	.674	.596	**.814**	.717	.481/.583/.423
MULAN + Angle	.728/.943	.655	.589	.751	.727	.480/.561/.404
MULAN + Contact	.722/.939	**.686**	**.599**	.738	.656	**.489**/.592/.428
MULAN + Foldseek	.730/.942	.633	.571	.801	.736	.479/.584/.430
MULAN + Coordinates	.712/.947	.684	.588	.797	**.755**	.487/.585/.420
ESM2 + Foldseek	**.735**/.947	.664	.564	.801	.733	**.498**/**.593**/**.431**

heads as output modules that add structural knowledge to MULAN embeddings via the structure-related loss functions. In all experiments, we follow the same training procedure as with MULAN.

Firstly, we conduct loss-only experiments without structure inputs. For this purpose, we finetune ESM2 8M with additional structure features prediction heads (ESM2 + Angle / ESM2 + Contact). The results of the evaluation are presented in Table 3 in the first section with ESM2. According to the results, using the structure information in the loss function is not sufficient for improvement on downstream tasks: only metrics for HumanPPI increased significantly.

Secondly, we evaluate MULAN with the extra structure features prediction heads (MULAN + Angle / MULAN + Contact). Moreover, we experiment with the Foldseek embedding layer as another source of structure inputs that can be passed to our model. We try both a combination of the Structure Adapter with a Foldseek embedding (MULAN + Foldseek) and the Foldseek embedding layer alone (ESM2 + Foldseek). The results are shown in the second part of Table 3. According to them, there is no clear evidence of superiority of one approach over another, we did not notice a consistent improvement. Thus, we decided to keep MULAN architecture as simple as possible and not to use additional heads. Also, we keep only the Structure Adapter as an input structure processing module. We do it because the use of the Foldseek embedding layer reduces the quality of Thermostability and Fluorescence prediction.

3.2. Contribution of sequence and structure modalities

Since MULAN has access to both sequence and structure angle data, we aim to analyze the contribution of both modalities. We show MULAN results with completely masked structural information (MULAN sequence-only experiment) and with the completely masked sequences (MULAN structure-only experiment) in Table 4. The results show that input protein structure angles highly influence the quality of MULAN embeddings, resulting in even lower quality for a sequence-only MULAN compared to the initial ESM2 8M model. The same is true for the structure-only scenario: the structure alone is not enough for good protein representations. This experiment shows the importance of both modalities and the use of the structural information by our model.

3.3. Learning rate strategies

We used the same learning rate for all parameters of MULAN during training. However, we initialize the whole protein encoder from the ESM2 pre-trained checkpoint, while the Structure Adapter is newly initialized. This fact suggests the possibility of using a smaller learning rate for ESM2 modules

Table 4. Contribution of different modalities in MULAN-ESM2 S performance

Model name	Localization 10-cl. / binary	Thermo-stability	Fluore scence	Metal Ion Binding	HumanPPI	GO CC / MF / BP
	acc ↑ / AUC ↑	SCC ↑	SCC ↑	AUC ↑	AUC ↑	F_{max} ↑
MULAN-ESM2 S	.732/.948	.674	.596	.814	.717	.481/.583/.423
sequence-only	.698/.945	.628	.564	.700	.777	.430/.363/.329
structure-only	.332/.546	.397	.100	.487	.569	.334/.177/.261

Table 5. Comparison of the performance of MULAN-ESM2 S with different learning rate (lr) strategies. If two learning rates are reported, the lowest corresponds to ESM2 modules, while the highest – for the Structure Adapter. For each section of the table best results are shown in bold

Model name	Localization 10-cl. / binary	Thermo-stability	Fluore scence	Metal Ion Binding	HumanPPI	GO CC / MF / BP
	acc ↑ / AUC ↑	SCC ↑	SCC ↑	AUC ↑	AUC ↑	F_{max} ↑
lr $5 \cdot 10^{-4}/5 \cdot 10^{-5}$	.716/.936	.674	.581	.743	**.732**	.493/.555/.407
lr $1 \cdot 10^{-3}/1 \cdot 10^{-4}$	.727/.944	**.675**	.575	.785	.680	**.496**/.578/**.423**
lr $1 \cdot 10^{-4}$ for all	**.732/.948**	.674	**.596**	**.814**	.717	.481/**.583**/**.423**

compared to the Structure Adapter in order not to harm the pre-trained weights a lot. This idea was suggested and shown its effectiveness in the work of Zhang et al. [19], where they have a similar combination of randomly initialized and pre-trained modules. We follow the suggested setup and decrease the learning rate for ESM2 modules by a factor of 10. According to the results, for MULAN there is no clear benefit of using the reduced learning rate for ESM2 modules (see Table 5), so we decided to keep the learning rate constant for all MULAN modules to reduce the number of used hyperparameters.

3.4. Additional experiments

3.4.1. Addition vs concatenation of embeddings

MULAN uses the structure bias from the Structure Adapter in a manner of positional embeddings: these structural embeddings are added to the main amino acid embeddings from the ESM2 model. However, one may concatenate these embeddings instead of summing up. This approach leads to different objectives used for amino acid embeddings (MLM) and structure embeddings (MLM for angle restoration). Also, concatenation leads to an increase in the length of the content passed to the Transformer model, causing significant memory and time overheads. The results of the experiment with the concatenation of embeddings did not show any benefit compared to the base setup.

3.4.2. Importance of angle masking

In our pre-training strategy, both amino acids and corresponding angle vectors are masked together. However, there are two other options that we have tested: independent masking of angles and residue letters and no angle masking at all. These experiments have shown worse results than the base approach. We explain it with the fact that residue letters and their angle vectors are connected. For example, if the letter is masked, and the angle vector has no side chain torsion angles defined, then the range of possible outcomes decreases from all 20 amino acids to only two: Glycine and Alanine. The opposite also holds: the known residue letter helps to restore the corresponding residue angle vector or at least the number of residue angles. Thus, we keep the joint masking strategy to force MULAN to learn as much information as possible.

4. Structural awareness of MULAN

According to the results from Table 6, all MULAN models demonstrate the awareness of the protein secondary structure. They surpass similar-sized ESM2 models by a large margin. Moreover, even a small MULAN utilizes the knowledge about the protein structure better than structure-aware ProstT5 and PST with more than a billion parameters. We do understand that the correct information about the secondary structure can be derived from angle inputs as well as from the Foldseek tokens used by SaProt or direct secondary structure types used by ESM3. Hence, this experiment is done only to demonstrate that MULAN actively uses the 3D structure.

5. Computational requirements comparison

We perform an analysis of the time and memory requirements for different large PLMs and SPLMs. We measure the inference time required for one forward pass as well as the amount of required VRAM on the Nvidia Tesla V100 GPU with 16Gb of VRAM. We take approximately the longest possible protein that can be handled on this GPU by ESM3: UniProt protein Q07009 with 702 residues, which is present in the test set of the GO annotation downstream task. We run 10 model inference runs and measure the average inference time for the protein.

The results are shown in Table 7. According to them, MULAN offers the best combination of quality and efficiency.

- Having the same computational costs as S-PLM, MULAN gives significantly better downstream results.
- MULAN requires 2 times less time and memory compared to PST, having similar downstream quality.
- MULAN requires only finetuning without training from scratch as in SaProt, ProstT5, and ESM3, having similar downstream quality.

Table 6. Comparison of the performance of PLMs on the secondary structure prediction task. The table is split into sections based on the model size. The best results for each section are in bold. For large models second best results are underlined

Model name	3-state, accuracy ↑			8-state, accuracy ↑		
	CASP12	TS115	CB513	CASP12	TS115	CB513
**Small models**						
ESM2 8M	.732	.798	.765	.602	.677	.623
MULAN-ESM2 S	**.894**	**.918**	**.895**	**.815**	**.854**	**.806**
**Medium models**						
ESM2 35M	.752	.828	.809	.619	.707	.669
MULAN-ESM2 M	.886	.901	.877	.789	.814	.769
SaProt AF 35M	.900	.924	.910	.805	.848	.817
MULAN-SaProt M	**.905**	**.927**	**.912**	**.807**	**.852**	**.818**
**Large models**						
ProstT5 1.2B	.858	.887	.891	.747	.800	.797
PST 1.1B	.853	.893	.880	.749	.802	.773
ESM3 1.4B	**.949**	**.969**	**.955**	**.921**	**.948**	**.923**
ESM2 650M	.821	.871	.867	.706	.772	.751
MULAN-ESM2 L	.867	.908	.890	.778	.833	.792
SaProt AF 650M	.926	.948	.945	.844	.897	.869
MULAN-SaProt L	.922	.949	.935	.841	.860	.870

Table 7. Comparison of the computational resources required for various large PLMs and SPLMs

Model name	# params	Inference time, ms	VRAM, MiB	From scratch
ESM2	650M	132	3274	-
SaProt AF	650M	126	3274	✓
MULAN-ESM2 L	652M	134	3368	✗
S-PLM	704M	103	3288	✗
PST	1.1B	283	6922	✗
Ankh large	1.2B	236	5292	-
ProstT5 (half)	1.2B	107	2974	✓
ProstT5 (full)	1.2B	217	5258	✓
ESM3	1.4B	355	13948	✓

6. Extended MULAN results

The performance gains induced by adding MULAN to ESM2 and SaProt models compared to the initial models used for MULAN initialization are shown in Table 8.

References

1. Ilya Loshchilov and Frank Hutter. Decoupled weight decay regularization. *arXiv preprint arXiv:1711.05101*, 2017.
2. Alexander Rives, Joshua Meier, Tom Sercu, Siddharth Goyal, Zeming Lin, Jason Liu, Demi Guo, Myle Ott, C Lawrence Zitnick, Jerry Ma, et al. Biological structure and function emerge from scaling unsupervised learning to 250 million protein sequences. *Proceedings of the National Academy of Sciences*, 118(15):e2016239118, 2021.
3. Philippe Gonzalez, Tommy Sonne Alstrøm, and Tobias May. On batching variable size inputs for training end-to-end speech enhancement systems. In *ICASSP 2023-2023 IEEE International Conference on Acoustics, Speech and Signal Processing (ICASSP)*, pages 1–5. IEEE, 2023.
4. José Juan Almagro Armenteros, Casper Kaae Sønderby, Søren Kaae Sønderby, Henrik Nielsen, and Ole Winther. DeepLoc: prediction of protein subcellular localization using deep learning. *Bioinformatics*, 33(21):3387–3395, 07 2017.
5. Christian Dallago, Jody Mou, Kadina E. Johnston, Bruce J. Wittmann, Nicholas Bhattacharya, Samuel Goldman, Ali Madani, and Kevin K. Yang. Flip: Benchmark tasks in fitness landscape inference for proteins. *bioRxiv*, 2021.
6. Anna Jarzab, Nils Kurzawa, Thomas Hopf, Matthias Moerch, Jana Zecha, Niels Leijten, Yangyang Bian, Eva Musiol, Melanie Maschberger, Gabriele Stoehr, et al. Meltome atlas—thermal proteome stability across the tree of life. *Nature methods*, 17(5):495–503, 2020.
7. Mingyang Hu, Fajie Yuan, Kevin K. Yang, Fusong Ju, Jin Su, Hui Wang, Fei Yang, and Qiuyang Ding. Exploring evolution-aware & -free protein language models as protein function predictors, 2022.
8. Xiao-Yong Pan, Ya-Nan Zhang, and Hong-Bin Shen. Large-scale prediction of human protein- protein interactions from amino acid sequence based on latent topic features. *Journal of proteome research*, 9(10):4992–5001, 2010.
9. Minghao Xu, Zuobai Zhang, Jiarui Lu, Zhaocheng Zhu, Yangtian Zhang, Ma Chang, Runcheng Liu, and Jian Tang. Peer: a comprehensive and multi-task benchmark for protein sequence understanding. *Advances in Neural Information Processing Systems*, 35:35156–35173, 2022.
10. Vladimir Gligorijević, P Douglas Renfrew, Tomasz Kosciolek, Julia Koehler Leman, Daniel Berenberg, Tommi Vatanen, Chris Chandler, Bryn C Taylor, Ian M Fisk, Hera Vlamakis, et al. Structure-based protein function prediction using graph convolutional networks. *Nature communications*, 12(1):3168, 2021.
11. Jin Su, Chenchen Han, Yuyang Zhou, Junjie Shan, Xibin Zhou, and Fajie Yuan. Saprot: Protein language modeling with structure-aware vocabulary. *bioRxiv*, pages 2023–10, 2023.
12. Karen S Sarkisyan, Dmitry A Bolotin, Margarita V Meer, Dinara R Usmanova, Alexander S Mishin, George V Sharonov, Dmitry N Ivankov, Nina G Bozhanova, Mikhail S Baranov, Onuralp Soylemez, et al. Local fitness landscape of the green fluorescent protein. *Nature*, 533(7603):397–401, 2016.

Table 8. Performance gains caused by adding MULAN to ESM2 and SaProt models compared to the initial models used for MULAN initialization. The quality is measured on 9 downstream tasks. We report average results across 5 random seeds used to initialize downstream models. We indicate the best results for each pair in bold. Statistically significant improvements with p-value < 0.05 are underlined. We report standard deviations across 5 runs in brackets.

Model name	Localization 10-cl. / binary	Thermo-stability	Fluore scence	Metal Ion Binding	HumanPPI	GO CC / MF / BP
	acc ↑ / AUC ↑	SCC ↑	SCC ↑	AUC ↑	AUC ↑	F_{max} ↑
ESM2 8M	.722(.001)/**.948**(.002)	.657(.025)	.585(.004)	.739(.009)	.657(.041)	**.481**(.004)/.532(.004)/.400(.001)
MULAN-ESM2 S	**.732**(.006)/**.948**(.001)	**.674**(.004)	**.596**(.003)	**.814**(.006)	**.717**(.015)	**.481**(.007)/**.583**(.002)/**.423**(.003)
ESM2 35M	.760(.006)/**.963**(.001)	.678(.006)	**.592**(.003)	**.783**(.008)	.668(.038)	.492(.003)/.608(.003)/.442(.001)
MULAN-ESM2 M	**.764**(.003)/**.963**(.001)	**.682**(.009)	.584(.005)	.771(.008)	**.787**(.009)	**.504**(.006)/**.628**(.002)/**.453**(.001)
Saprot AF 35M	.767(.003)/.960(.001)	**.695**(.004)	.634(.002)	.784(.008)	.703(.026)	**.499**(.010)/**.633**(.002)/.440(.003)
MULAN-SaProt M	**.769**(.005)/**.962**(.001)	**.695**(.007)	**.638**(.004)	**.797**(.007)	**.766**(.012)	.496(.006)/.632(.001)/**.444**(.001)
ESM2 650M	**.818**(.004)/**.968**(.001)	.691(.005)	.595(.005)	**.786**(.005)	.748(.015)	**.516**(.018)/**.676**(.002)/**.479**(.002)
MULAN-ESM2 L	.815(.003)/**.968**(.001)	**.702**(.003)	**.614**(.002)	.766(.012)	**.847**(.007)	.515(.010)/**.676**(.003)/**.479**(.002)
SaProt AF 650M	.840(.003)/**.972**(.001)	**.711**(.002)	**.668**(.001)	.779(.007)	.712(.015)	**.535**(.008)/**.661**(.001)/.467(.002)
MULAN-SaProt L	**.841**(.004)/**.972**(.001)	.709(.005)	.667(.001)	**.798**(.010)	**.766**(.009)	.530(.009)/.660(.002)/**.468**(.004)

13. Roshan Rao, Nicholas Bhattacharya, Neil Thomas, Yan Duan, Peter Chen, John Canny, Pieter Abbeel, and Yun Song. Evaluating protein transfer learning with tape. *Advances in neural information processing systems*, 32, 2019.
14. Kim T Simons, Rich Bonneau, Ingo Ruczinski, and David Baker. Ab initio protein structure prediction of casp iii targets using rosetta. *Proteins: Structure, Function, and Bioinformatics*, 37(S3):171–176, 1999.
15. John Moult, Krzysztof Fidelis, Andriy Kryshtafovych, Torsten Schwede, and Anna Tramontano. Critical assessment of methods of protein structure prediction (casp)—round xii. *Proteins: Structure, Function, and Bioinformatics*, 86:7–15, 2018.
16. Yuedong Yang, Jianzhao Gao, Jihua Wang, Rhys Heffernan, Jack Hanson, Kuldip Paliwal, and Yaoqi Zhou. Sixty-five years of the long march in protein secondary structure prediction: the final stretch? *Briefings in bioinformatics*, 19(3):482–494, 2018.
17. James A Cuff and Geoffrey J Barton. Evaluation and improvement of multiple sequence methods for protein secondary structure prediction. *Proteins: Structure, Function, and Bioinformatics*, 34(4):508–519, 1999.
18. Hannes Stärk, Christian Dallago, Michael Heinzinger, and Burkhard Rost. Light attention predicts protein location from the language of life. *Bioinformatics Advances*, 1(1):vbab035, 2021.
19. Zuobai Zhang, Chuanrui Wang, Minghao Xu, Vijil Chenthamarakshan, Aurélie Lozano, Payel Das, and Jian Tang. A systematic study of joint representation learning on protein sequences and structures, 2023.
